# Carotid Stump Pressure and Contralateral Internal Carotid Stenosis Ratio During Carotid Endarterectomies: 1D-0D Hemodynamic Simulation of Cerebral Perfusion

**DOI:** 10.3400/avd.oa.20-00166

**Published:** 2021-03-25

**Authors:** Sohei Matsuura, Toshio Takayama, Changyoung Yuhn, Marie Oshima, Takuro Shirasu, Takafumi Akai, Toshihiko Isaji, Katsuyuki Hoshina

**Affiliations:** 1 Division of Vascular Surgery, Department of Surgery, Graduate School of Medicine, The University of Tokyo, Tokyo, Japan; 2 Department of Mechanical Engineering, The University of Tokyo, Tokyo, Japan; 3 Interfaculty Initiative in Information Studies, The University of Tokyo, Tokyo, Japan

**Keywords:** carotid stenosis, carotid endarterectomy, circle of Willis, cerebral collateral circulation

## Abstract

**Objective:** We selectively place carotid shunting when ipsilateral mean stump pressure is less than 40 mmHg during carotid endarterectomy (CEA). This study aimed to assess the validity of our selective shunting criterion by 1D-0D hemodynamic simulation technology.

**Materials and Methods:** We retrospectively reviewed 88 patients (95 cases) of CEA and divided them into two groups based on the degree of contralateral internal carotid artery (ICA) stenosis ratio, which was determined as severe when the peak systolic velocity ratio of the ICA to the common carotid artery was ≥4 by carotid duplex ultrasonography. Patients with severe stenosis or occlusion in contralateral ICA were classified as hypoperfusion group, and those without such contralateral ICA obstruction were classified as control group.

**Results:** Perioperatively, the mean carotid stump pressures were 33 mmHg in hypoperfusion group and 46 mmHg in the control group (P=0.006). We simulated changes in carotid stump pressure according to the changes in the contralateral ICA stenosis ratio. 1D-0D simulation indicated a sharp decline in carotid stump pressure when the contralateral stenosis ratio was >50%, while peripheral pressure of the middle cerebral arteries declined sharply at a ≥70% contralateral stenosis ratio. At this ratio, the direction of the ipsilateral cerebral arterial flow became inverted, the carotid stump pressure became dependent on the basilar artery circulation, and the ipsilateral middle cerebral artery became hypoperfused.

**Conclusion:** Our clinical and computer-simulated results confirmed the validation of our carotid shunting criterion and suggested that contralateral ICA stenosis ratio over 70% is a safe indication of selective shunting during CEA.

## Introduction

Carotid arterial stenosis is a major source of stroke and is one of the leading causes of death and disability worldwide. A carotid endarterectomy (CEA) is an established surgical procedure designed to prevent stroke from carotid arterial stenosis.^1,2)^ Nevertheless, CEA itself can cause ischemic stroke as a consequence of carotid artery clamping during the procedure, especially when the contralateral blood supply is insufficient.^3)^ Carotid stump pressure (CSP) is used to measure contralateral blood supply.^4)^ Stenosis or occlusion of the contralateral carotid artery results in lower CSP during carotid clamping.^5–8)^

Although the circle of Willis (CoW) is the main collateral circulation network of the brain, the A1 segment of the anterior cerebral artery (next to the internal carotid artery [ICA]) may be the strongest single vessel predictor of CSP.^9)^ Lownie et al. showed that the intraoperative systemic mean arterial blood pressure in combination with preoperative angiography findings accurately predicted CSP.^8)^

A 1D-0D simulation can return hemodynamic quantities in the CoW during CEA, including CSP for various stenosis ratios of the contralateral ICA. This study aims to determine the relationship between CSP and the contralateral ICA stenosis ratio from simulation results using real patient data. We hypothesized that there would be a strong relationship between CSP and contralateral ICA stenosis.

## Materials and Methods

### Patients

We retrospectively reviewed the medical records of 88 patients and 95 cases of CEA performed at our institution between August 1998 and January 2019 (**Table 1**). All cases were included. The experimental protocol and informed consent were approved by our Institutional Review Board (IRB No. 3316-(4)). Patient anonymity was ensured, and all patients gave written informed consent for publication of this report.

**Table 1:** Table 1 Patient characteristics

	Hypoperfusion (N=18)	Control (N=77)	P-value
Background			
Age (years)	70±1.8	71±0.86	0.80
Men	16 (89%)	66 (86%)	0.72
Symptomatic	14 (78%)	52 (68%)	0.40
Hypertension	13 (72%)	61 (79%)	0.52
Diabetes mellitus	10 (56%)	33 (43%)	0.33
Dyslipidemia	10 (56%)	48 (62%)	0.60
Smoker	17 (94%)	62 (81%)	0.16
Ischemic heart disease	15 (83%)	42 (55%)	0.025
Chronic kidney disease	7 (39%)	29 (39%)	0.99
Perioperative characteristics			
Mean CSP±SD (mmHg)	33±4.1	46±2.0	0.006
Carotid shunt, n (%)	17 (94%)	41 (53%)	0.0013
Operative time±SD (min)	272±24	265±12	0.81
Perioperative stroke, n (%)	0 (0%)	1 (1.4%)	0.66
30-day death, n (%)	0 (0%)	0 (0%)	NA

CSP: carotid stump pressure; SD: standard deviation

According to our previous study,^10)^ ICA stenosis ratios were classified into four categories by carotid duplex ultrasonography: (1) “no stenosis” when the ICA peak systolic velocity (PSV) was lower than 125 cm/s, (2) “moderate (50–69%) stenosis” when the ICA PSV was 125 cm/s or higher, (3) “severe (70–99%) stenosis” when the PSV ratio of ICA to the common carotid artery was 4 or higher, and (4) “occlusion” when the ICA revealed no flow.

Our general indication of CEA was either symptomatic moderate/severe ICA stenosis or asymptomatic severe stenosis. We routinely monitored the intraoperative cerebral perfusion by near-infrared spectroscopy and measured the ipsilateral CSP just after clamping. Stump pressure was measured as follows: We first clamped the ICA, common carotid artery (CCA), and external carotid artery in this order; we then declamped the ICA, followed by puncturing the CCA with a 22 G needle connected to pressure transducer. A carotid shunting tube was placed when the mean CSP was less than 40 mmHg to maintain the ipsilateral cerebral perfusion.

There were 75 men and 13 women included in the study. Their ages ranged from 46 to 87 years, with a mean age of 70.9±7.54 years. Seven patients underwent bilateral CEAs and these 14 cases were individually analyzed. We refer to individual CEAs as “cases.” Eighteen cases with severe stenosis or occlusion in the contralateral ICA were considered the “hypoperfusion group,” and the other 77 were considered the “control group.”

### 1D-0D hemodynamic simulation

The 1D-0D cardiovascular model^11,12)^ was used to simulate the change of CSP in accordance with the degree of contralateral ICA stenosis. This model consisted of the 1D model, which solves blood flow in large arteries as pulse wave propagation, and the 0D model, which considers the effects of the remaining circulatory system. The 0D stenosis model^13)^ was integrated into the 1D model to adequately evaluate the pressure loss across the stenosis. These reduced-order models, i.e., 1D-0D models, have been widely validated both in vitro and in vivo^14–16)^ and used as a means to answer specific clinical questions.^17,18)^

**Figure 1:**
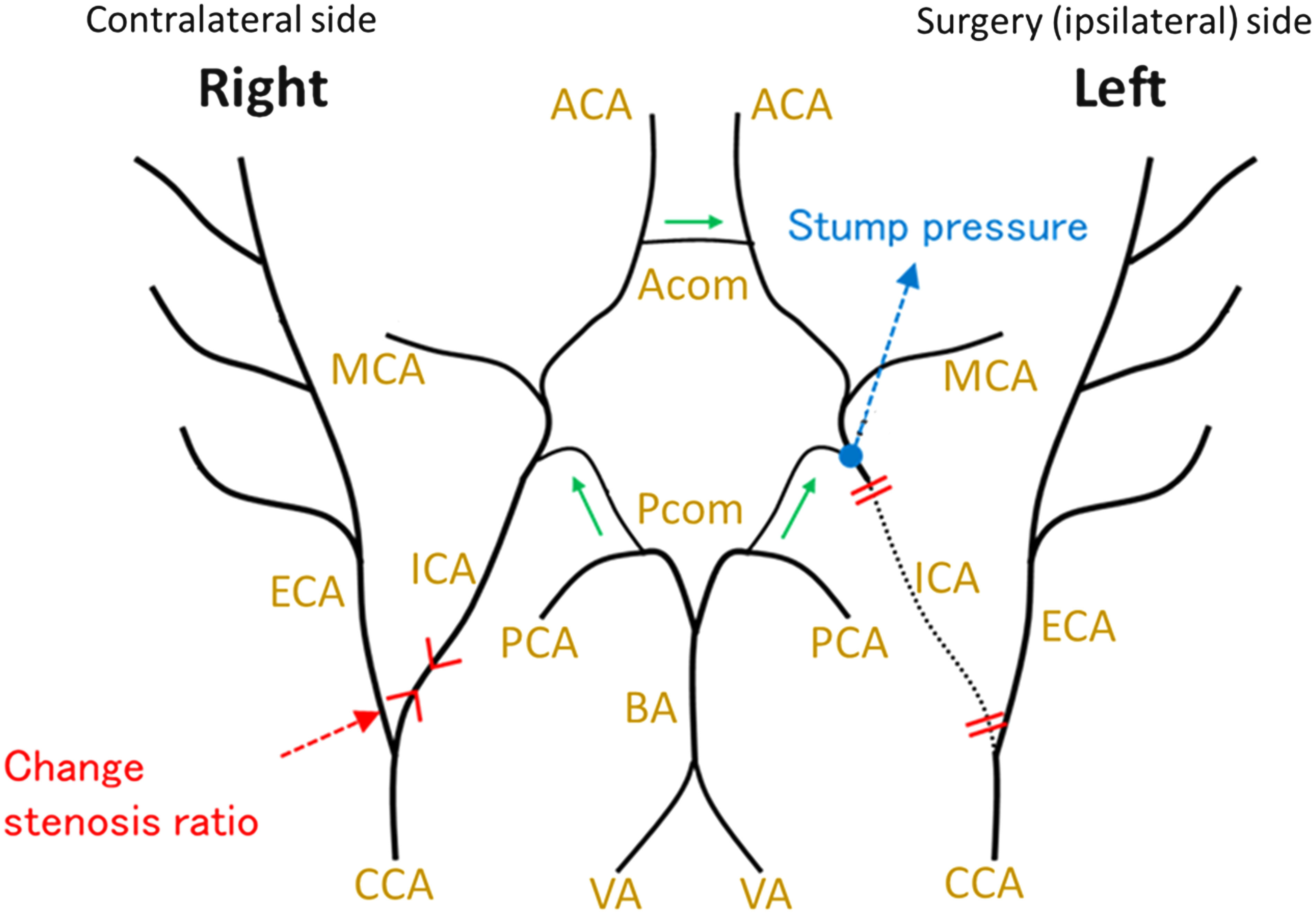
Fig. 1 Schematic of the simulation setup; the CEA was set to the left side. We changed the contralateral carotid stenosis ratio and observed the change in CSP. The contralateral (left) ICA lesion length was set to 10 mm. The simulated CSP was calculated according to the contralateral ICA stenosis ratio.

CEA: carotid endarterectomy; CSP: carotid stump pressure; ICA: internal carotid artery

To simplify the simulation setup, we created an imaginary 70-year-old male patient with a mean arterial pressure of 90 mmHg. The model parameters were assigned based on population-averaged data,^11,12)^ with consideration given to age-associated changes,^19)^ and further calibrated to yield the target value of the mean arterial pressure.^20)^ As illustrated in **Fig. 1**, we postulated that left-sided CEA was performed with total blockade of ipsilateral ICA blood flow. We set a 10 mm length of the contralateral ICA stenosis, starting from 10 mm above the carotid bifurcation. We varied the stenosis ratio of the vessel lumen from 0 to 100% with 10% increments.

In real-life situations, the cerebral blood flow is maintained at a constant level through the cerebral autoregulation system, even when blood pressure drastically changes. To incorporate this effect into the simulation, we obtained the flow rate–pressure relationship of the cerebral arteries by fitting a smooth curve to experimental data available in the literature.^12)^ Using this relationship, we calculated a converged solution of cerebral blood flow under clamping and with the existence of stenosis. Liang et al. provided a more detailed description of this process.^12)^

### Statistical analysis

Statistical analyses were performed using JMP Pro 14 statistical software (SAS Institute Japan, Tokyo, Japan) to identify statistically significant differences between the control and hypoperfusion groups. Continuous variables were compared using the Student’s t-test, and proportions were compared using Fisher’s exact test. P-values <0.05 were considered statistically significant.

## Results

### Patient data analysis

Patient characteristics are shown in **Table 1**. There were no significant differences between the hypoperfusion and control groups with respect to average age, symptom rates, smoking, diabetes, hypertension, dyslipidemia, or chronic kidney disease with an estimated glomerular filtration rate ≤50 mL/min/1.73 m^2^. The rate of coronary arterial disease was significantly higher in the hypoperfusion group (P=0.025).

Perioperative factors are shown in **Table 1**. For the intraoperative factors, the mean CSP was significantly lower in the hypoperfusion group than in the control group (33.3 mmHg vs. 46.2 mmHg, P=0.006). Therefore, the carotid shunt rate was also significantly different between the two groups (94% in hypoperfusion group vs. 53% in the control group, P=0.0013).

There were no perioperative strokes or deaths in the hypoperfusion group, but there was one perioperative stroke in the control group (P=0.65). None of the patients died by the 30th postoperative day in either group. In the case of postoperative stroke, the patient developed right hemiparesis 8 days after left CEA; magnetic resonance image revealed a left corona radiata infarction. Symptoms quickly resolved and the patient was discharged on postoperative day 12.

There were no statistically significant differences between the groups in terms of overall survival (OS) (P=0.50) or disease-free survival (DFS) (P=0.50).

### Hypothetical hemodynamic change in response to contralateral carotid stenosis

**Figure 2:**
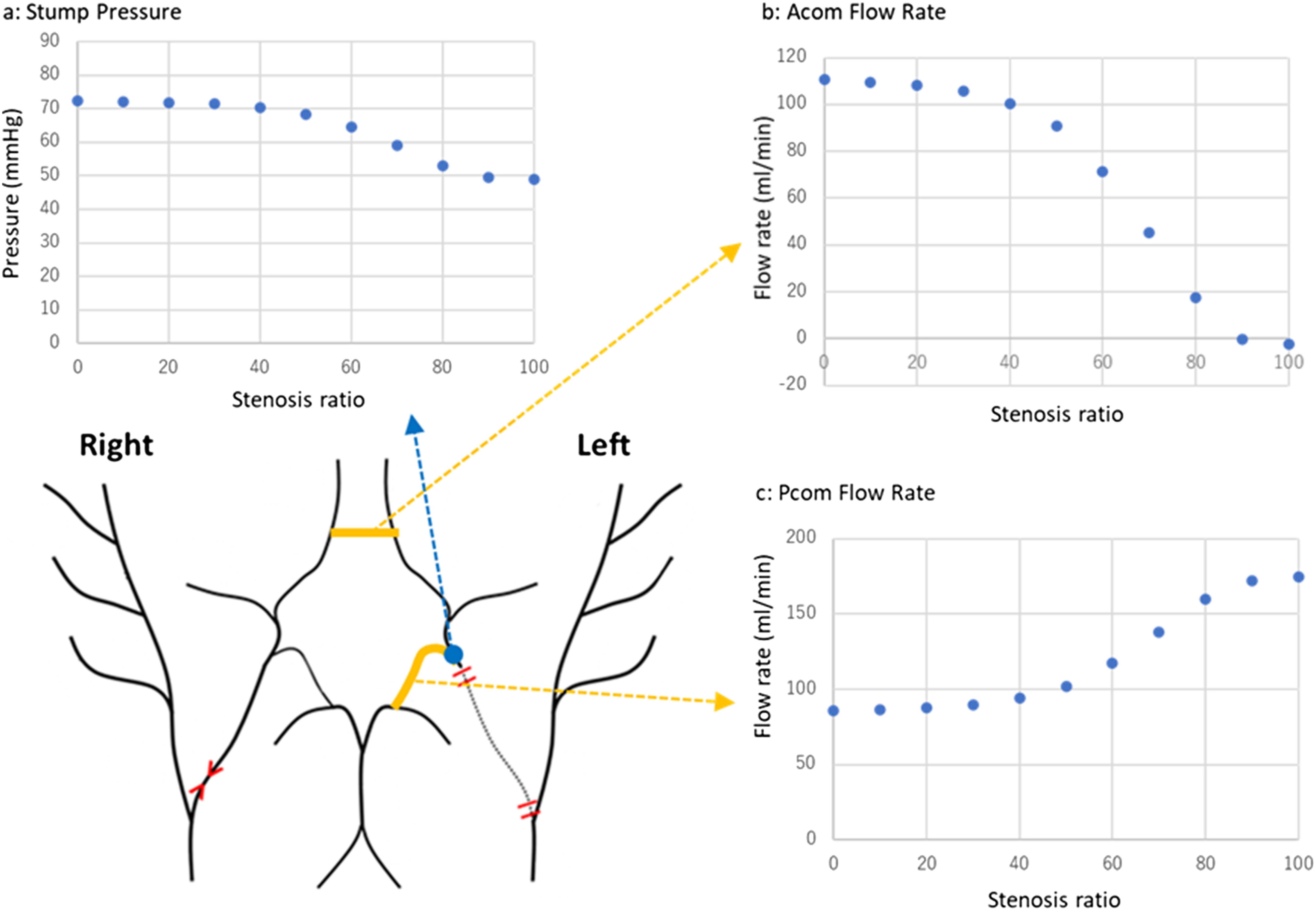
Fig. 2 The CSP change and changes in flow rates of the communicating arteries. The CSP was constant when the contralateral stenosis was under 40%; it declined linearly when the contralateral stenosis ratio was over 50% (**a**). The Acom flow rate decreased linearly when the contralateral stenosis ratio was over 50% (**b**). The ipsilateral Pcom flow rates increased linearly when the contralateral stenosis ratio was over 50% (**c**).

Our simulation model demonstrated that the CSP was maintained at the same level as long as the contralateral stenosis ratio was below 40%. The CSP declined linearly when the contralateral stenosis ratio was above 40%. Of note, the CSP fell below 60 mmHg when the contralateral stenosis ratio was 70% (**Fig. 2a**).

Acom: anterior communication artery; Pcom: posterior communicating artery; CSP: carotid stump pressure

In the analysis of flow rate and flow direction, the flow rate of the anterior communicating artery (Acom) began to decrease linearly when the contralateral stenosis ratio was 50%, and the flow disappeared when the contralateral stenosis ratio was 90% (**Fig. 2b**). The flow rates of bilateral posterior communicating arteries (Pcoms) began to increase linearly when the contralateral stenosis ratio was 50% (**Fig. 2c**).

**Figure 3:**
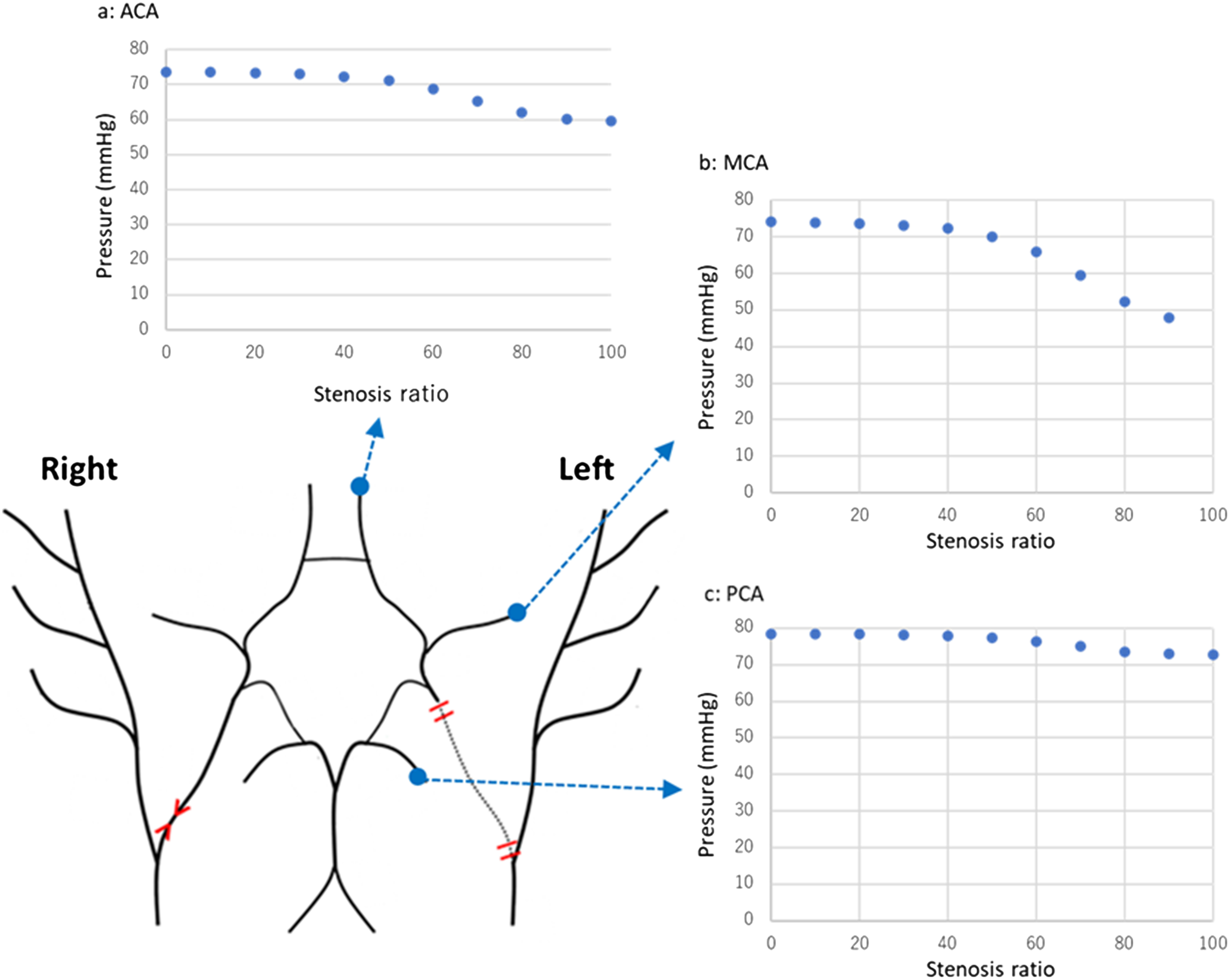
Fig. 3 Peripheral pressures of the ipsilateral cerebral arteries. The pressures of both ipsilateral ACA and PCA remained steady, regardless of the contralateral stenosis ratio (**a**, **c**), whereas the pressure of the ipsilateral MCA began to decline linearly when the contralateral stenosis ratio was 60% (**b**).

ACA: anterior cerebral artery; MCA: middle cerebral artery; PCA: posterior cerebral artery

The pressure of the ipsilateral anterior cerebral artery (ACA) and posterior cerebral artery (PCA) remained steadily above 60 mmHg and 70 mmHg for each, regardless of the ratio of contralateral stenosis (**Figs. 3a** and **3c**). The pressure of the ipsilateral middle cerebral artery (MCA) began to decline linearly when the contralateral stenosis ratio was 60% (**Fig. 3b**).

**Figure 4:**
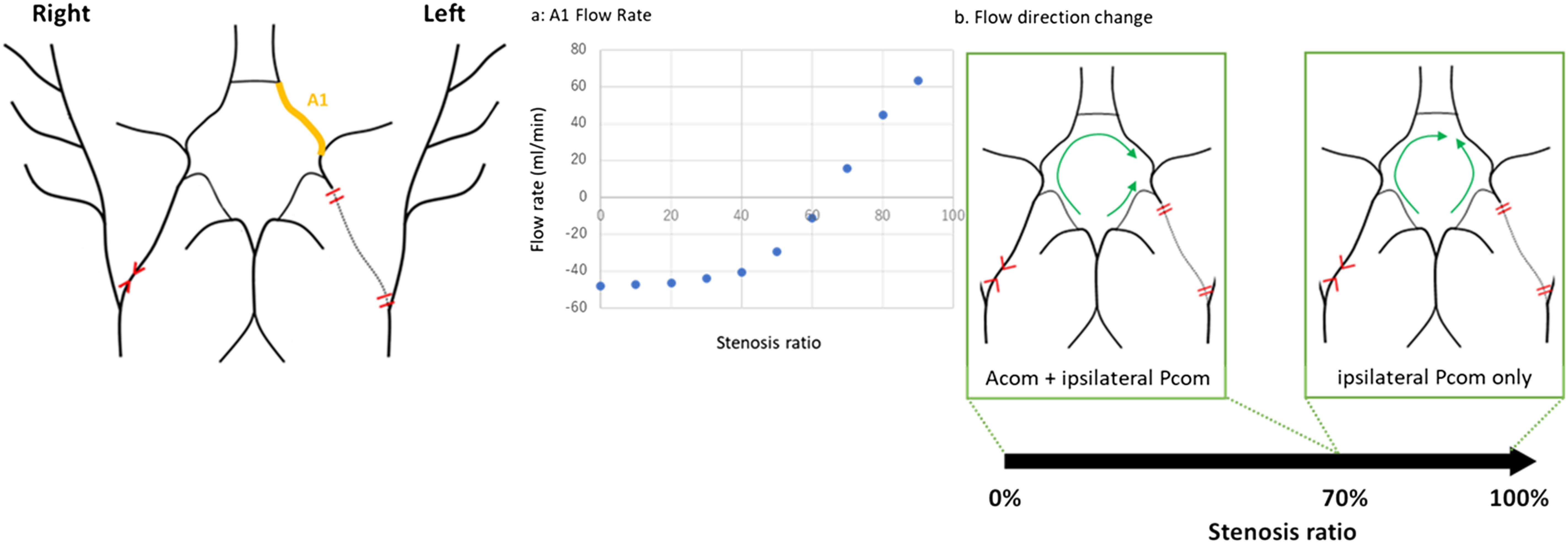
Fig. 4 Changes in flow rate and flow direction of the ipsilateral ACA (A1 segment). The ipsilateral ACA flow direction inverted when the contralateral stenosis ratio was over 70% (**a**). The ipsilateral MCA was perfused by both the Acom and ipsilateral Pcom when the contralateral stenosis ratio was under 70%. In addition, it was perfused only by the ipsilateral Pcom when the contralateral stenosis ratio was over 70% (**b**).

ACA: anterior cerebral artery; Acom: anterior communication artery; Pcom: posterior communicating artery

The flow direction in the ipsilateral ACA A1 segment inverted when the contralateral stenosis ratio was over 70% (**Fig. 4a**). The ipsilateral MCA was perfused by both Acom and ipsilateral Pcom when the contralateral stenosis ratio was under 70%, while it was perfused only by ipsilateral Pcom when the contralateral stenosis was over 70% (**Fig. 4b**).

## Discussion

We found that patients with high-grade (over 70%) contralateral ICA stenosis had a higher background incidence rate of ischemic heart disease. These patients also had lower stump pressure and a higher carotid shunting rate as intraoperative factors. In contrast, their perioperative and long-term outcomes were equivalent to those of patients without high-grade contralateral ICA stenosis. This finding suggests that CEA can be safely carried out in patients with high-grade contralateral ICA stenosis if intraoperative brain perfusion is properly maintained.

Using our simulation model, we aimed to determine the optimal time to apply the shunt. Our simulation setup consisted of two critical features: (1) the contralateral ICA stenosis rate was manipulated (0% to 100%) while the ipsilateral ICA was clamped, and (2) the other arterial network, including the CoW, remained constant regardless of the contralateral ICA stenosis ratio. These features enabled us to determine the critical point of contralateral ICA stenosis.

When the contralateral ICA stenosis ratio exceeded 50%, the simulation results showed that the ipsilateral ICA stump pressure and Acom flow rate began to decrease linearly, while the Pcom flow began to increase linearly. This finding suggested that collateral flow from the vertebrobasilar artery became dominant to compensate for the anterior brain circulation at this contralateral ICA stenosis ratio. When the contralateral ICA stenosis ratio exceeded 60%, the ipsilateral MCA pressure began to drop linearly, while the ipsilateral ACA and PCA pressures remained almost constant up to 100% occlusion of the contralateral ICA. This suggests that the MCA is particularly vulnerable to contralateral ICA stenosis.

When the contralateral ICA stenosis ratio exceeded 70%, our simulation demonstrated that the flow direction of the ipsilateral ACA A1 segment inverted. In other words, the ipsilateral A1 segment blood flowed toward the ipsilateral MCA through the Acom when the contralateral ICA stenosis ratio was under 70%; however, it began to flow away from the MCA when the contralateral ICA stenosis ratio was over 70%. The net effect of this flow inversion resulted in a significant decrease in MCA blood flow, explaining the vulnerability of the MCA to contralateral ICA stenosis. Based on these results, we considered the critical point of contralateral ICA stenosis to be 70%, consistent with our clinical findings.

In practice, the CSP is often used to measure the cerebral perfusion during carotid clamping, and the execution of temporary shunting is decided based on the CSP value in combination with findings from electroencephalography or other continuous brain monitoring modalities. CEA under local anesthesia can be the best way to monitor the brain because the patient’s consciousness level immediately indicates the tolerance against carotid clamping.^21)^ The GALA study demonstrated that both general and local anesthesia were equally safe for CEA.^22)^ In reality, however, the fear of possible sudden coma may cause patients to prefer general anesthesia.^23)^ Therefore, the method of anesthesia is flexible based on the surgeon’s or patient’s choice; however, all of our CEAs have been performed under general anesthesia.

Although there are no absolute CSP criteria for carotid shunting under general anesthesia,^24)^ a mean CSP above 60 mmHg suggests sufficient cerebral perfusion, and selective shunting may be unnecessary.^7,25)^ In other words, CSP above 60 mmHg might indicate a “safety zone.” Our result also supports the appropriateness of the 70% contralateral ICA stenosis critical point for carotid shunting, as this is the point where the CSP drops beyond the “safety zone,” and the collateral pathway to the ipsilateral MCA changes.

Overall, we found that the contralateral carotid artery is the dominant collateral pathway during carotid clamping; this suggests that there is theoretically little need for carotid shunting if the contralateral carotid artery is free from severe stenosis or occlusion.

Despite the fact that there is little evidence regarding the threshold value of cerebral perfusion pressure, 60 mmHg might be a safe threshold for preventing cerebral ischemia.^26)^ Furthermore, our findings suggest that cerebral perfusion in the region of the ipsilateral MCA may become insufficient at the 70% degree of contralateral carotid stenosis. This result also supports using the 70% degree of contralateral carotid stenosis as an indication for selective shunting.

### Limitations

One of the limitations of the study was that the simulation did not consider the compensatory remodeling of vessels for decreased blood flow due to the presence of contralateral carotid stenosis. Nevertheless, other investigators using magnetic resonance angiography in patients who had minor disabling cerebral infarction found that those with severe ICA stenosis showed no significantly increased CoW vessel diameters.^27)^ Although there are many CoW variations,^28)^ they were not considered in this study. One of the most critical limitations was that our simulation model did not include vertebral artery/basilar artery/Pcom diseases, even though such situations are often seen in clinical settings. We ignored whether patients had complete CoW for the purpose of simplification of the simulation.

A carotid shunting tube was placed when the mean CSP was less than 40 mmHg or oxygen saturation value measured by near-infrared spectroscopy dropped more than 5% within 1 min after clamping (we have reported good correlation between these two indications^29)^).

## Conclusion

Our clinical data supported the efficacy of selective shunting in cases of severe contralateral ICA stenosis. Our simulation suggested that the ipsilateral MCA perfusion significantly decreased at the 70% ratio of contralateral ICA stenosis, which was considered a critical point. Overall, these results suggest that selective shunting should be considered in cases where the contralateral ICA stenosis ratio is more than 70%.

## Data Availability

All data are available in the process of review.
